# Feasibility and effectiveness of a targeted diabetes prevention program for 18 to 60-year-old South Asian migrants: design and methods of the DH!AAN study

**DOI:** 10.1186/1471-2458-12-371

**Published:** 2012-05-23

**Authors:** Everlina MA Vlaar, Irene GM van Valkengoed, Vera Nierkens, Mary Nicolaou, Barend JC Middelkoop, Karien Stronks

**Affiliations:** 1 Department of Public Health, Academic Medical Center, University of Amsterdam, Amsterdam, The Netherlands; 2 Department of Public Health and Primary Care, Leiden University Medical Center, Leiden, The Netherlands; 3 Public Health Service, The Hague, The Netherlands

## Abstract

**Background:**

South Asian migrants are at particularly high risk of type 2 diabetes. Previous studies have shown that intensive lifestyle interventions may prevent the onset of diabetes. Such interventions have not been culturally adapted and evaluated among South Asians in industrialized countries. Therefore, we have set up a randomized controlled trial to study the effectiveness of a targeted lifestyle intervention for the risk of type 2 diabetes and cardiovascular risk factors among 18 to 60-year-old Hindustani Surinamese (South Asians) in The Hague, the Netherlands. Here we present the study design and describe the characteristics of those recruited.

**Methods:**

Between May 18, 2009 and October 11, 2010, we screened 2307 Hindustani Surinamese (18–60 years old) living in The Hague. We sent invitations to participate to those who had an impaired fasting glucose of 5.6-6.9 mmol/l, an impaired glucose tolerance of 7.8-11.0 mmol/L, a glycated hemoglobin level of 6.0% or more and/or a value of 2.39 or more for the homeostasis model assessment of estimated insulin resistance. In total, 536 people (56.1% of those eligible) participated. People with a higher level of education and a family history of type 2 diabetes were more likely to participate. The control and intervention groups were similar with regard to important background characteristics. The intervention group will receive a culturally targeted intervention consisting of dietary counseling using motivational interviewing and a supervised physical activity program. The control group will receive generic lifestyle advice. To determine the effectiveness, a physical examination (anthropometrics, cardiorespiratory test, lipid profile, and measures of oral glucose tolerance, glycated hemoglobin, and insulin) and interview (physical activity, diet, quality of life, and intermediate outcomes) were carried out at baseline and will be repeated at 1 year and 2 years. The process and the costs will be evaluated.

**Discussion:**

This trial will provide insight into the feasibility and effectiveness of a targeted, intensive, lifestyle intervention for the risk of type 2 diabetes and cardiovascular risk factors among 18 to 60-year-old South Asians.

**Trial registration:**

Dutch Trial Register: NTR1499

## Background

Type 2 diabetes mellitus (DM) is one of the most common chronic diseases in the industrialized countries [1]. The burden of DM is expected to increase due to factors such as aging, urbanization, and the increasing prevalence of obesity and physical inactivity [1].

South Asian migrants and their offspring living in industrialized countries (henceforth “South Asians”) are at particularly high risk of DM [1-4]. In the Netherlands for example, the prevalence among the Hindustani Surinamese, who are of South Asian origin, is about four times as great as that among the ethnic Dutch in the same age group [4]. South Asians are not only younger at presentation, they are also at high risk of developing complications [3-6]. Hence, prevention of new DM cases and DM-related morbidity among South Asians potentially leads to an important health gain [7].

Previous studies have shown that intensive lifestyle interventions prevent the onset of DM among older populations with pre-diabetes [8-10]. Yet, while it seems particularly relevant, such intensive lifestyle interventions have not been implemented among South Asians in industrialized countries. There are two reasons. First, information about the feasibility and effectiveness of such interventions is lacking. This is particularly important, as South Asians are less likely to participate in intervention programs than their European counterparts [7,11,12]. Second, these interventions have not culturally targeted South Asians, while cultural adaptations likely have great influence on the effectiveness of interventions among specific populations [13,14].

Therefore, we want to study the effectiveness of a targeted, intensive, lifestyle intervention concerning the risk of DM and cardiovascular risk factors among 18 to 60-year-old Hindustani Surinamese (South Asians) in The Hague, the Netherlands. We will also evaluate the process and costs of the intervention to determine whether the targeted intervention program was feasible and implemented as planned. This paper a) outlines the protocol for the recruitment and a 2-year randomized controlled trial (RCT) and b) describes an analysis of the participation in the RCT and the baseline characteristics of the participants.

## Methods/design

In this paper, we describe the protocol of the RCT. The original protocol was adjusted during the study period because the recruitment period was extended, but the end date of the study was fixed. The changes in the original protocol are described in the Online Supplement (Additional file 1).

### Study design

**Figure 1:**
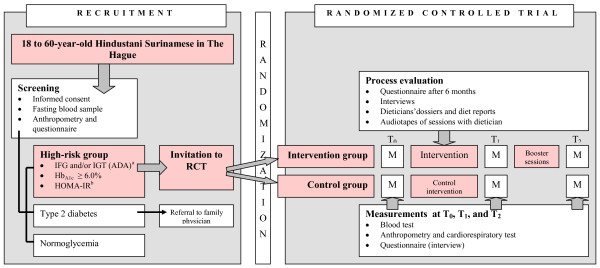
** Design of the DH!AAN study.** Legend: ^a^FPG of 100–125 mg/dl (5.6–6.9 mmol/l) and/or 2-h postload glucose of 140–199 mg/dl (7.8–11.0 mmol/l), ^b^HOMA-IR ≥ 2.39. ADA, American Diabetes Association; FPG, fasting plasma glucose; IFG, impaired fasting glucose; IGT, impaired glucose tolerance; Hb_A1c_, glycated hemoglobin; HOMA-IR, homeostasis model assessment of estimated insulin resistance; M, measurements; RCT, randomized controlled trial.

This study was set up as an RCT with one intervention group and one control group (Figure 1). We invited potential participants for a screening to determine their risk of DM. If DM was suspected, the person concerned was referred to the family physician for further diagnostic testing and treatment. Those who appeared to be at “high risk of DM” were invited to participate in the RCT. We used a computer-generated randomization list to randomly assign invitees who gave their informed consent to the intensive lifestyle intervention or the control group. Family members (*n* = 38), that is, participants belonging to the same household, defined as having the same postal code and house number, were assigned to the same program.

The main goal was to compare the risk of DM and several cardiovascular risk factors in the intervention group and the control group over time. The primary outcomes are changes in weight, mean blood glucose concentration, and behavioral change [diet and physical activity (PA)]. The secondary outcomes are cardiovascular risk factors, the use of primary health care and intermediate behavioral outcomes. Therefore, measurements were carried out at baseline and will be repeated after 1 and 2 years. The Medical Ethics Committee of the Academic Medical Center of Amsterdam, The Netherlands, has approved the study.

### Recruitment of the study population

#### Study population and invitation procedure

The study population consisted of Hindustani Surinamese persons. The term “Hindustani Surinamese” refers to people of South Asian ancestral origin and their offspring who migrated to the Netherlands via Suriname. The Hindustani Surinamese are the descendants of the labourers from North India - Uttar Pradesh, Uttaranchal, and West Bihar – who were indentured between 1873 and 1917. The two large migration waves of Hindustani Surinamese to the Netherlands were caused mainly by the political situation in Suriname. The first wave took place at the time of the independence of Suriname in 1975, and the second wave, at the time of Desi Bouterse’s coup in February 1980 [15].

**Figure 2:**
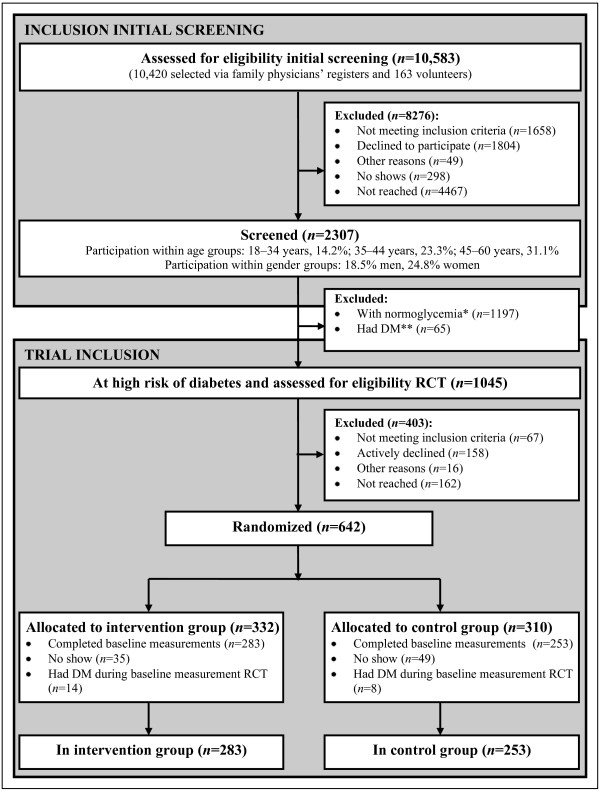
** Flow diagram from eligibility assessment to inclusion in the trial.** Legend: *Including n = 129 classified as normoglycemia during initial screening due to original protocol and n = 7 classified as normoglycemia during the baseline measurement and prior to randomization due to original protocol (Additional file 1); **Including n = 2 classified as DM during the baseline measurement and prior to randomization due to original protocol (Additional file 1); “High risk of DM” refers to having a fasting plasma glucose of 100–125 mg/dl (5.6 – 6.9 mmol/l) and/or 2-h postload glucose of 140–199 mg/dl (7.8–11.0 mmol/l); DM, type 2 diabetes mellitus; RCT, randomized controlled trial.

We selected 10,583 Hindustani Surinamese (South Asians), aged 18–60 years, from 48 family physician lists in The Hague by means of name analysis. We chose the age range of 18 to 60 years because of the high prevalence of DM among 35 to 44-year-old South Asians in the Netherlands [4]. The researcher, the family physician or the practice nurse, and a trained research assistant of Hindustani Surinamese origin verified the names. We excluded people known to have DM and pregnant women (Figure 2). We chose to recruit via the family physician because of evidence that ethnic minority groups in the Netherlands in general appear to have confidence in their family physician ( [16,17] and unpublished data). Moreover, we assumed that working through health care providers would facilitate future implementation. 

The family physicians sent each potential participant an invitation letter with a reply card that could be returned if further contact was unwanted. Family members and other volunteers (*n* = 163) could also make an appointment for the screening. We culturally tailored the invitation by using the colors of the Surinamese flag and a Hindustani eye in the logo of the study, by citing a key figure encouraging participation, and by using the study name ‘DH!AAN’ – an acronym that means “attention” in Sarnami Hindustani, the Surinamese variant of Hindi. Moreover, we used plain language in the letter and leaflet, and we adjusted the risk information about DM to the Hindustani Surinamese population.

We sent a written reminder to invitees who had not responded to the invitation within 2 weeks and invited them to make an appointment. The reminder also said that if no response was received (no appointment or reply card) within 1 week, the invitee would be contacted by telephone. The study team phoned those who had not responded on at least three occasions (attempts varying in the afternoon and in the evening). If no telephone number was available (as was the case for 24.1% of the potential participants) or potential participants could not be reached, we sent a second written reminder asking people to contact us. This intensive recruitment strategy was tested during the pilot study and proved to be feasible and to achieve a higher response rate than only written invitations and reminders (unpublished data).

#### Initial screening

The initial screening took place between May 18, 2009 and November 11, 2010. All participants (n = 2307) in the screening were asked to donate a fasting blood sample. We asked them to answer a brief questionnaire, which asked about their self-identified ethnicity (including the country of birth of the participants and of their parents), education level, their own DM status and that of first- and second-degree relatives, and previous medical diagnoses. Trained research staff carried out physical examinations in a standardized protocol in which they measured weight, height, and blood pressure (Table 1). All measurements were obtained twice.

**Table 1:** Measurements among participants in the RCT

**Measurements**	**Method or sample used**	**Screening -recruitment period**	**RCT**					
			**Baseline (T**_**0**_**)**	**6 Months**	**1 year (T**_**1**_**)**		**2 years (T**_**2**_**)**	
					**SC**	**HV**	**SC**	**HV**
**I. Laboratory assessments**								
1.Fasting plasma glucose, Hb_A1c_, insulin	Fasting venous blood sample	x	x		x	d	x	d
2.2-h Postload glucose	2-h postload glucose	b	x		x		x	
3.Triglycerides, HDL, LDL, cholesterol, creatinine, hs-CRP	Fasting venous blood sample		x		x		x	
4.Albumin and creatinine (urine)	Fasting urine sample	x	x		x	x	x	x
5.Blood sample storage (serum, EDTA)	Fasting venous blood sample	b	c				x	
**II.Anthropometry**								
6.Weight, height, blood pressure (Omron M5-1)	SOP: twice, blood pressure measured 5 times	x	x		x	x	x	x
7. Waist and hip circumferences, fat percentage (Omron 500)	SOP: twice	b	x		x		x	
**III. Behavior**								
8.PA (SQUASH [22] adapted to SA [23])	Questionnaire (interview)		x		x	x	x	x
9.Diet (based on guideline for healthful diet)	Questionnaire (interview)	x	x		x	x	x	x
10.Smoking [24]	Questionnaire (interview)	x	x		x	x	x	x
11.Alcohol [25]	Questionnaire (interview)	x	x		x	x	x	x
**IV. Other measurements**^**a**^								
12.Ethnicity, household information	Questionnaire	x						
13.Education, income, insurance, religion [26]	Questionnaire (interview)	education	x		x	x	x	x
14.Physical health (cardiovascular risk profile; [27])	Questionnaire (interview)	f	g		g	g	g	g
15.Cardio respiratory test (Chester STEP test; [28,29])	SOP		x		x		x	
16.Perceived quality of life (SF-15; [30,31])	Questionnaire (interview)		x		x	x	x	x
17.Distress (part of 4DSQ; [32])	Questionnaire (interview)		x		x	x	x	x
18.Risk perception ( [33] and our own questionnaire)	Questionnaire (interview)		x	x	x	x	x	x
19.Attitude, knowledge, social support, self-efficacy, and stages of change in PA and diet	Questionnaire (interview)		x		x	x	x	x
20.Perception of one’s own body	Questionnaire (interview)		x		x	x	x	x
**V. Process**								
21.Dose received (participation in intervention components, frequency, leaflet, satisfaction)	Questionnaire (interview)			x				
**VI. Costs**								
1.Use of health care	Questionnaire (interview)		x		x	x	x	x
2.Costs of screening and lifestyle intervention	Questionnaire (interview)	h	x		x	x	x	x

Legend: ^**a**^Questionnaire developed for the study; b – participants until April 19^th^, 2010 (n = 968); c – participants since April 19^th^, 2010; d – only *Hb*_*A1c*_ measurement; f – heart disease, hypertension, high cholesterol, artritis and diabetes; g – heart failure and stroke; h – only costs screening; *SC*, screening; *HV*, home visit; *SOP*, standard operating procedures; *PA*,physical activity; *HDL*, high-density lipoprotein; *hs-CRP*, highly sensitive C-reactive protein; *LDL*, low-density lipoprotein; *PA*, physical activity; *SA*, South Asian(s); *SQUASH,* Short Questionnaire to Assess Health-Enhancing Physical Activity; *4DSQ*, Four-Dimensional Symptom questionnaire.

The participants who were invited and screened between May 18, 2009 and April 18, 2010 (*n* = 968) took an oral glucose tolerance test (OGTT; 75 g). The physical examination for these participants also included measurements of body fat and waist and hip circumference. Moreover, four blood samples were collected for storage (Table 1).

#### Inclusion and exclusion criteria

If the person’s fasting glucose was impaired [a fasting plasma glucose (FPG) level of 5.6-6.9 mmol/l], glucose tolerance was impaired (2-h postload of 7.8-11.0 mmol/l), the hemoglobin (Hb)_A1c_ was 6.0% or more, and/or the value for the homeostasis model assessment of estimated insulin resistance (HOMA-IR) was 2.39 or more [18-21], then the person was considered “at high risk of DM” and was eligible for the RCT. The threshold for HOMA-IR is based on the median value for HOMA-IR of the first 349 participants in the screening and is comparable to the median among people with DM of 2.4, as Matthews et al. (1985) find ( [21] and unpublished data).

We asked potential participants for their consent (oral and written) and excluded anyone who was already involved in a lifestyle program, was pregnant, had a chronic disease that made participation in the intervention impossible, and/or used drugs interfering with plasma glucose levels. We evaluated this condition on a case-by-case basis.

#### Response and participation

##### Participation in the initial screening

Of all those who received an invitation, 2307 (21.8%) participated in the screening (Figure 2): 18.5% of the men and 24.8% of the women. The participation rate was higher among those aged 45–60 years (31.1%) than among those aged 18–34 years (14.2%) or 35–44 years (23.3%). The net participation rate was 25.8% (2307 of those eligible (n = 8925)).

##### Participation in the randomized controlled trial

In total, 536 people (283 intervention group; 253 control group) were included in the RCT (Figure 2). Of all the people considered at high risk of DM (*n* = 1045), 158 (15.1%) actively declined to participate. Moreover, 89 people (8.5%) were found not eligible; 67 prior to the randomization and 22 during the baseline measurement because of suspected DM. The net participation rate in the trial was 56.1% (536 of those eligible (n = 956)).

The characteristics of the participants, as measured in the initial screening, were comparable with the characteristics of the nonparticipants, with the exception of family history of DM and education (Table 2). Participants more often reported having a family member with DM than nonparticipants (chi-squared test, *p* = 0.043). Moreover, the participants had a somewhat higher level of education than the nonparticipants (Mann–Whitney *U* test, *p* = 0.023). Nonparticipants seemed to have a higher blood pressure and were more likely to be classed as having elevated fasting glucose, but this was not significant.

**Table 2:** Determinants of participation for those eligible for the trial

	**All eligible people**	**Participants**	**Nonparticipants**	Significance*
	***n*** **= 956**^**^**^	***n*** **= 536**	***n*** **= 420**	
Median age in years	44 (36–51)	45 (37–52)	44 (35–51)	0.13
Men (%)	468 (49.0)	265 (49.4)	203 (48.3)	0.73
18–34 years (%)	212 (22.2)	109 (20.3)	103 (24.5)	
35–44 years (%)	280 (29.3)	159 (29.7)	121 (28.8)	
45–60 years (%)	464 (48.5)	268 (50.0)	196 (46.7)	
Education (%)				0.02**
Elementary^a^	126 (13.6)	61 (11.7)	65 (16.1)	
Intermediate^b^	645 (69.8)	364 (70.0)	281 (69.6)	
University or equivalent^c^	153 (16.6)	95 (18.3)	58 (14.4)	
Relatives with DM (%)^d^	685 (73.7)	398 (76.2)	287 (70.3)	0.04**
Median weight in kg	73.5 (64.8–82.0)	73.3 (65.0–82.1)	73.8 (64.0–82.1)	0.90
Median BMI in kg/m^2^	26.8 (24.4–29.9)	26.8 (24.5–29.7)	26.7 (24.1–30.3)	0.98
Overweight: ≥ 23 kg/m^2^ (%)^e^	419 (44.9)	246 (46.9)	173 (42.3)	0.17
Obesity: ≥ 27.5 kg/m^2^ (%)^e^	412 (44.1)	227 (43.2)	185 (45.2)	0.54
Median SBP in mm Hg	128.0 (119.0–38.0)	127.0 (118.0–137.0)	129.0 (120.0–0.0)	0.07
Median DBP in mm Hg	83.0 (76.0–90.0)	82.0 (76.0–90.0)	84.0 (76.0–92.0)	0.06
Hypertension (%)^f|^	62 (38.4)	196 (37.1)	166 (40.1)	0.34
Median FPG (mmol/l)	5.3 (4.9–5.7)	5.3 (4.9–5.7)	5.4 (5.0–5.7)	0.61
Elevated FPG^†^(%)	332 (34.8)	174 (32.5)	158 (37.9)	0.08
Median Hb_A1c_ in percentage	5.7 (5.4–6.0)	5.7 (5.4–6.0)	5.7 (5.4–5.9)	0.36
Elevated Hb_A1c_: ≥ 6.0% (%)	238 (25.1)	138 (25.8)	100 (24.2)	0.57
Median HOMA in mmol/mol	3.2 (2.6–4.4)	3.2 (2.5–4.3)	3.3 (2.7–4.4)	0.36
HOMA-IR: ≥ 2.39 (%)	800 (84.0)	453 (84.5)	347 (83.4)	0.65

Determinants measured during initial screening only; only determinants measured in total population are shown Data are *n* (percentages, calculated excluding missings), means (SD) if normally distributed or medians (IQRs) if not normally distributed *^*All people labeled as ‘at high risk of diabetes’ *(n* = 1045) minus people who were found not eligible for the trial (*n* = 67 prior to randomization and *n* = 22 because of suspected DM during baseline measurement) *p calculated with chi-squared test (binary data), independent *t*-test (continuous data), and Mann–Whitney *U* test (ordinal/nominal data and not normal distributed data).

^**^p < 0.05 – chi-squared test (Relatives with DM)/Mann–Whitney *U* test (Education).

^a^Primary education or less.

^b^Low vocational training, lower secondary education, intermediate vocational training and higher secondary education.

^c^Higher vocational training or university.

^d^First and second grade.

^e^BMI of 23–27.5 kg/m^2^ was considered overweight; a BMI of 27.5 kg/m^2^ or more, obesity [48].

^f^SBP ≥140 mm Hg and/or of DBP ≥ 90 mm Hg or if hypertension medication.

^g^American Diabetes Association definition: fasting glucose value of 100–125 mg/dl (5.6–6.9 mmol/l).

*BMI*, body mass index; *DBP*, diastolic blood pressure; *DM*, type 2 diabetes mellitus; *FPG*, fasting plasma glucose; *Hb*_*A1c*_, glycated hemoglobin; *HOMA-IR*, homeostasis model assessment of estimated insulin resistance; *IQR*, interquartile range; *SBP*, systolic blood pressure.

#### Baseline characteristics of the participants

The median age of the participants was 45 years [interquartile range (IQR): [37-52] and 265 participants (49.4%) were men (Table 3). Most participants had an intermediate education level and had relatives in the first or second degree with DM. The median body mass index (BMI) was 26.9 kg/m^2^ (IQR: 24.6–29.7), and the mean waist circumference was 95.0 cm (SD: 10.9) for men and 90.7 cm (SD: 10.5) for women. Moreover, the mean FPG level was 5.3 mmol/l (SD: 0.5), the median 2-h postload plasma glucose level was 5.8 mmol/l (IQR: 4.8–6.9) and the median Hb_A1c_ level was 5.7% (IQR: 5.4–5.9). No significant differences were found between the intervention group and the control group (Table 3). 

**Table 3:** Baseline characteristics of the participants

	**Participants**	**Intervention group**	**Control group**	Significance*
	***n* = 536**	***n* = 283**	***n* = 253**	
Median age in years	45 (37–52)	45 (37–52)	44 (37–51)	0.49
Men (%)	265 (49.4)	136 (48.1)	129 (51.0)	0.50
Education (%)				0.36
Elementary^a^	61 (11.7)	27 (9.9)	34 (13.7)	
Intermediate^b^	364 (70.0)	192 (70.6)	172 (69.4)	
University or equivalent^c^	95 (18.3)	53 (19.5)	42 (16.9)	
Family with DM (%)^d^	398 (76.2)	214 (77.8)	184 (74.5)	0.37
Median BMI in kg/m^2^	26.9 (24.5–29.6)	26.9 (24.7–29.5)	27.0 (24.4–29.8)	0.90
Mean waist circumference in cm				
Men	95.0 (10.9)	95.0 (10.5)	95.0 (11.4)	0.99
Women	90.7 (10.5)	90.4 (10.6)	91.1 (10.4)	0.60
Median SBP in mm Hg	125.5 (117.0–37.3)	125.7 (117.5–7.5)	125.0 (116.5–36.5)	0.53
Mean DBP in mm Hg	83.0 (10.0)	83.3 (9.9)	82.7 (10.2)	0.51
Hypertension (%)^e|^	200 (38.6)	105 (38.3)	95 (38.9)	0.89
Plasma glucose in mmol/l				
Mean FPG	5.3 (0.5)	5.3 (0.6)	5.3 (0.5)	0.77
Median 2-h postload glucose	5.8 (4.8–6.9)	5.9 (5.0–7.0)	5.5 (4.8–6.9)	0.12
Median Hb_A1c_ in %	5.7 (5.4–5.9)	5.7 (5.4–5.9)	5.6 (5.4–5.9)	0.31
Median HOMA-IR	3.1 (2.2–4.1)	3.1 (2.2–4.2)	3.0 (2.3–4.1)	0.91
Median insulin in pmol/l	12.9 (9.6–17.3)	13.4 (9.6–17.5)	12.8 (9.6–17.1)	0.86
Mean LDL in mmol/l	3.2 (0.9)	3.2 (0.8)	3.2 (0.9)	0.83
Median HDL in mmol/l)	1.2 (1.0–1.4)	1.2 (1.1–1.4)	1.2 (1.0–1.4)	0.27

Data are given as *n* (percentages, calculated excluding missings), means (SD) if normally distributed or medians (IQRs) if not normally distributed.

*p is given as calculated with the chi-squared test (binary data), independent *t*-test (continuous data), or Mann–Whitney *U* test (ordinal/nominal data and not normally distributed data).

^a^Primary education or less.

^b^Low vocational training, lower secondary education, intermediate vocational training and higher secondary education.

^c^Higher vocational training and university.

^d^First and second grade.

^e^Systolic blood pressure ≥ 140 mm Hg and/or diastolic blood pressure ≥ 90 mm Hg or if hypertension medication.

*BMI*, body mass index; *DBP*, diastolic blood pressure; *DM*, type 2 diabetes mellitus; *FPG*, fasting plasma glucose; *Hb*_*A1c*_, glycated hemoglobin; *HDL*, high-density lipoprotein; *HOMA-IR*, homeostasis model assessment of estimated insulin resistance; *IQR*, interquartile range; *LDL*, low-density lipoprotein; *SBP*, systolic blood pressure; *SD*, standard deviation.

### Intervention group: The design of the lifestyle intervention

The aim of the lifestyle intervention is to meet the current guidelines for diet and PA [34,35]. We used data from previous qualitative and quantitative research within the Department of Public Health to adjust the intervention design, which was based on the design of the intervention in the SLIM study, to the situation in The Hague and the Hindustani Surinamese population [36-38].

The intervention consists of culturally targeted lifestyle counseling and a supervised PA program (exercise on prescription). Trained dieticians will conduct the lifestyle counseling, which consists of individual sessions and an optional program comprising a family session and cooking classes. The combination of diet and PA is often used in intensive lifestyle programs and appears to be more effective in preventing DM than diet or PA alone [8,9,39]. To target the diabetes prevention program to the Hindustani Surinamese population, we have added both surface and deep structure adaptations to make the intervention attractive, appropriate, and ultimately more effective [14]. The colors and key figures in the leaflet are examples of using the superficial characteristics of a cultural group; they can be seen as surface-structure adaptations [14]. Deep structure adaptations are adaptations targeted to factors that influence health behavior [14]. Paying specific attention to social pressure and support is an example of a deep structure adaptation in this study.

#### Individual lifestyle counseling

The individual lifestyle counseling consists of six to eight sessions in the 1st 6 months, followed by 3 to 4 booster sessions in the next 1.5 years, and it aims at both diet and PA. We use motivational interviewing (MI) as a counseling strategy, taking into account dietary habits and cultural norms regarding diet and PA. Motivational interviewing can be defined as “a client-centered, directive method for enhancing intrinsic motivation to change by exploring and resolving ambivalence” [40]. Motivational interviewing is an upcoming intervention methodology, and the evidence for MI for lifestyle advice seems to be promising [41,42].

The six dieticians who will conduct the lifestyle counseling are familiar with the Hindustani Surinamese culture and dietary habits. The medical psychologist who trained the dieticians in MI is an expert in MI [43]. Special attention will be paid to possible barriers and stimulating factors that are specific to the Hindustani Surinamese population. As recommended, we will provide continued support for MI to achieve optimal results in appliance and effects [44].

#### Family session and cooking classes

Besides the individual sessions, participants will also be offered a family session with the dietician. The purpose of this session is to decrease the social pressure to eat unhealthily and to increase the social support for a healthful lifestyle for the family. This entails providing product information and discussing the importance of a healthful lifestyle and how to increase support.

The participants will be offered two cooking classes to increase their self-efficacy in skills that they can learn for adjusting traditional dishes in a more healthful way. They will be encouraged to bring spouses or close relatives with them to increase their knowledge and attitude towards healthful diet.

#### Exercise on prescription

The intervention group will be offered “exercise on prescription” (EoP), a 20-week supervised PA program, which has previously been described [45-47]. The participants can choose between aquarobics and fitness, offered in men-only or women-only groups [45,46]. Trained PA coaches will monitor and supervise the participation. At the end of the 20 weeks, a final evaluation will take place, at which time the lifestyle advisor will provide information about low-budget sport locations.

### Control group

The participants in the control group will be invited to join two group sessions led by student dieticians (after baseline measurements and after 6 months). The student dieticians will provide generic information about diabetes, discuss the current guidelines for diet and PA, list methods for achieving the recommended PA, and present some cases. In the second session, one respondent will be given the opportunity to become a case study. In addition, the control group will receive two flyers (at 3 months and 9 months) with simple, generic lifestyle advice. Both flyers offer participants a chance to phone in during preset hours to obtain personal advice from a dietician.

### Data collection

For both the intervention group and the control group, the baseline (T_0_) and subsequent measurements after 1 (T_1_) and 2 years (T_2_) of the trial include blood sampling [fasting and 2-hour OGTT (glucose load 75 g)], a structured face-to-face interview, and a physical examination. These actions all took place, and will take place again, in one central health care center in The Hague. The participants will receive a questionnaire after 6 months as part of the process evaluation. Participants who do not fill in the questionnaire at 6 months will be asked to complete it at T_1_ in the health care center. Table 1 shows the measures recorded at baseline and to be recorded again after 6 months, 1 year, and 2 years. Participants who were not reached and any participants who indicate that they intend to quit the trial at T_1_ or T_2_ will be offered a brief home visit instead of the more extensive screening at the health care center (Table 1).

#### Laboratory assessments

Plasma glucose, insulin, and Hb_A1c_ levels and other biomarkers will be assessed (Table 1). All laboratory assessments will be carried out according a standardized protocol at the General Practice Laboratory Foundation, Etten-Leur, The Netherlands. At different time points, material is stored at −80°C for future analyses (Table 1).

#### Physical examination

Trained research staff carries out the physical examinations in a standardized protocol. A Seca mechanical scale (Seca 761, Seca, Hamburg, Germany) is used to weigh the participants (wearing light clothing) to the nearest 500 g. Height is recorded to the nearest 0.01 m on a Seca portable stadiometer (Seca 214, Seca, Hamburg, Germany), and waist and hip circumferences are determined to the nearest 0.01 m with a tape measure. A bioelectrical impedance analysis (OMRON BF500, Omron Healthcare, Hoofddorp, The Netherlands) is used to determine body fat to the nearest 0.1%. All anthropometric measurements are obtained twice – we allowed differences between two measurements of 2 cm, 2 kg, or 2% – and the means will be used for analysis.

The participant is in the seated position when the blood pressure (Omron M5-1, Omron Healthcare, Hoofddorp, The Netherlands) is measured on the nondominant arm supported at heart level. At most, five measurements are taken. We calculate the mean from the first two measurements with less than 5 mm Hg difference in both systolic and diastolic blood pressure [49]. We assess cardiorespiratory fitness with the Chester STEP test [28,29].

#### Structured face-to-face interview

##### Behavioral outcomes

We will assess PA with the Short Questionnaire to Assess Health-enhancing Physical Activity (SQUASH), supplemented with ethnicity-specific activities [22,23]. Dietary intake will be determined with questions based on the national guideline for a healthful diet, supplemented with questions about specific dietary habits of the Hindustani Surinamese population [34,38]. We will assess fruit, vegetables, rice, and bread consumption with multi-item measurements to determine the quantity and frequency (*n* = 3, *n* = 2, *n* = 2, and *n* = 11, respectively), and two single-item questions will address the use of breakfast and the regularity of the meals. We will also measure the current smoking and alcohol consumption (Table 1) [24,25].

##### Intermediate outcome measures

Health-related quality of life will be measured with short form (SF)-12 and three extra emotional status questions from SF-36 [30,31]. We will measure distress with the 16 distress questions of the Four-Dimensional Symptom Questionnaire (distress, depression, anxiety, and somatization) [32].

We will measure personal vulnerability with a three-item personal vulnerability score [33] and risk perception with 12 statements about the participant’s perceived risk of diabetes in connection with overweight, current smoking, and/or unhealthy eating. The statements are based on general risk factors and information from focus group discussions [37,50].

We will determine attitudes towards PA and diet directly with questions about importance and joy regarding several aspects of PA and diet (e.g., PA in leisure time; breakfast; fruit, vegetable and whole grain intake). We will assess attitude towards PA indirectly with seven items about the possible consequences of increasing PA (e.g., “If I increase my PA, I will look better”).

We will ask whether the spouse, other family members, or close relatives encourage the respondent to be physically active (*n* = 3). Moreover, we will measure overall support towards the targeted aspects of diet (*n* = 7).

We will measure self-efficacy expectations towards diet change and dealing with specific PA barriers with five items about PA and seven items about aspects of diet [focus group discussions; unpublished data].

The stage of change towards PA and diet will be measured with the algorithm described in the Transtheoretical Model [51]. This includes three scales regarding motivation to change, described as: unmotivated to change diet and PA within 6 months, motivated to change at least parts of the diet and PA within 6 months, and prepared to change within 1 month [52].

##### Background characteristics

We will assess physical health, religion, and socioeconomic status (as indicated by educational level, source of income, and net family income) using questions similar to those of the SUNSET study [26,27].

### Activities to encourage participation in the data collection

We will send all participants the DH!AAN study newsletter twice a year in order to inform them of the progress of the study and to encourage continued participation. The participants were rewarded with a gift coupon of €5 to €10 for their participation in the baseline measurements; this reward will be repeated for subsequent measurements. Moreover, a prize in the form of a trip to Suriname will be raffled to one of the participants of the T_1_ and T_2_ measurements.

### Statistical analysis

#### Size of the study population and the power calculation

The Online supplement (Additional file 1) describes the original power calculation. Here we calculate the power of the study to demonstrate differences in the main outcomes using the numbers of participants in the intervention and control groups.

A total of 536 participants were included in the RCT with a minimum of 253 participants per group. Previous studies found a difference in weight reduction of 1.5–3 kg (SD of 4) [10,53], and a difference in FPG of 0.2–0.35 mmol/l (SD of 0.7) [10,53]. Assuming a an alpha of 5%, and a dropout rate of 30%, we have a power of 86% or more to demonstrate a minimum difference in weight reduction of 1.25 kg and a change in glucose level of 0.2 mmol/l with our data.

#### Effectiveness of the intensive lifestyle intervention

Statistical analysis will be performed according to the intention-to-treat principle and, if necessary, corrected for differences in background characteristics between the intervention and control groups. To assess the effect of the intervention program on the risk of diabetes and cardiovascular risk factors after 1 and 2 years, we will calculate differences between the intervention and control groups in general linear mixed models (GLMM). If we observe a difference between the intervention and control groups, we will examine whether the magnitude of the effect is most likely due to other cardiovascular risk factors or behavioral change. We will also use GLMM to examine the influence of baseline variables and the degree of participation (e.g., adherence and the actual carrying out of the components of the intervention) in weight reduction and behavioral changes. We will use multilevel analyses to distinguish group effects (e.g., effect of dietician’s work) from individual effects.

#### Process evaluation

In the process evaluation we will investigate for each intervention component fidelity, dose received (exposure and satisfaction) and dose delivered [54]. Several measures will be used including a structured questionnaire at six months, semi-structured interviews with a sample of participants and non-participans, dieticians’ files and audiotapes of the counseling sessions of participants with dieticians.

We will measure fidelity with questions in the questionnaire and semi-structured interview about relevance, understandability, and satisfaction with each component of both lifestyle interventions. We will determine the level of MI applied from the audiotapes using One Pass, which is based on the Motivational Interviewing Skill Code scoring list [55]. We will assess the level of cultural sensitivity during the MI sessions with a scoring list specially designed for this study. We based it on a combination of the Seeleman and colleagues’ cultural sensitivity model and diverse existing scoring lists for cultural sensitivity [56-58].

We will measure measure the dose received (exposure) and the dose delivered by asking participants about their participation in each component of the intervention and analysing the dieticians’ registration files.

#### Evaluation of the costs

We developed questions in collaboration with a health economist to determine the costs. We asked participants to note their travel time, mode of transportation, travel costs, and hours of work missed for the screening in the recruitment phase and at T_0_, but will only ask about any change in transportation at T_1_ and T_2_ (Table 1). We will measure costs for the intervention program in the questionnaire at 6 months; for example, the time invested, the cost of the change of diet, and the cost of participating in an exercise program (e.g., sportswear; Table 1). Moreover, to measure whether the screening and intervention affected the use of primary health care, we will ask participants about their use of certain health care, laboratory assessments, and medication at T_0_, T_1_, and T_2_ (Table 1).

## Discussion

Our study is unique in terms of population (South Asians in industrialized countries), age (young population), the mode of recruitment, and the culturally targeted design of the intervention. No studies have reported on the feasibility and potential effectiveness of an intensive lifestyle intervention in this population, while South Asians in industrialized countries are at high risk of DM and are therefore an important target group. Given this high risk of DM, we used broad criteria for inclusion. For instance, in contrast with previous studies, we did not restrict participation to those with overweight. We selected participants on the basis of high levels of Hb_A1c_ and HOMA-IR as these markers have been independently associated with diabetes risk [19,20,59,60]. Using Hb_A1c_ as a marker for DM and people at high risk of DM is in line with recent recommendations [19,61].

This strategy is illustrative for the design of our trial, which incorporates both pragmatic and explanatory elements according to the definitions of Roland and Togerson [62]. Because of this, we expect to be able to provide information how and why the intervention works, but also insight in whether this intervention may work in real life conditions.

Despite the lack of specific evidence, results from earlier studies suggest that DM screening and intervention among South Asians – with a culturally sensitive approach – may be effective and lead to a decrease in the burden of DM and risk of cardiovascular disease [7,13,63,64]. For instance, one study in India has reported an effect of a lifestyle intervention on the incidence of DM. However, given the substantial differences in context, their results are not generalizable to South Asians living in industrialized countries [64].

### Limitations

Our study has some limitations that should be mentioned. First, participation in the initial screening and at baseline was relatively low, despite our intensive and targeted recruitment strategy. The participation rate is often low in studies among ethnic minority groups in western countries [7,12,65,66]. Our initial screening had a higher participation rate than another study that was partially targeted to this population [7].

The low participation may be associated with selection bias. We cannot rule this out in our study. As in previous studies, participation in the initial screening was highest among women and older people [8,67,68]. Moreover, participation at baseline was greater among people with a higher education level and among those who reported having family with diabetes than among those who did not. This may be related to the fact that well-educated people or people who have a family history of DM are more motivated to engage in preventive behavior [66-69]. At the same time, other background characteristics of the participants and nonparticipants were comparable. Nevertheless, we should keep in mind that selection bias may affect the generalizability of our findings.

Another limitation of the study is that, during its course, we were forced to shorten the duration of the follow-up to 2 years, instead of maintaining the originally intended 3 years. As a consequence, the focus on the incidence of type 2 diabetes shifted to other outcome measures. We acknowledge that this limits the assessment of the effectiveness for the prevention of incident DM. However, we believe that our findings will still provide valuable insight into feasibility and will indicate the potential health gain that can be achieved.

Changes in several relevant outcomes, e.g., diet and physical activity, will be assessed by means of questionnaires. However, self-reported behavior may be subject to recall bias and social desirability [70]. This can be partly overcome if we combine these self-reported data with an assessment of more objective measures intended to provide insight into the effectiveness [71].

In summary, this study will contribute to the evidence base for lifestyle interventions for the prevention of DM. Specifically, the trial will provide insight into the potential effectiveness of a targeted intensive lifestyle intervention among 18 to 60-year-old South Asians in an industrialized country who form a population at high risk of DM. During the 2-year study we will assess the changes in a broad range of relevant outcomes. Moreover, we expect that the evaluation of the process and costs will provide important information about the feasibility of a culturally targeted lifestyle intervention among South Asians via general practices and other health care providers.

## Competing interests

The authors declare that they have no competing interests.

## Authors’ contributions

EV analyzed the data, contributed to the interpretation, and drafted the manuscript. IV, VN, and KS contributed to the design of the study and the interpretation of the data. They also reviewed and edited the manuscript. BM and MN gave advice for the design and interpretation and reviewed the manuscript. All authors read and approved the final manuscript.

## Pre-publication history

The pre-publication history for this paper can be accessed here:

http://www.biomedcentral.com/1471-2458/12/371/prepub

## Supplementary Material

Additional file 1 **Online supplement.** Changes to the original protocol of the randomized controlled trial [2,8,36,53,64]. Click here for file

## References

[B1] Wild S Roglic G Green A Sicree R King H Global prevalence of diabetes: estimates for the year 2000 and projections for 2030 Diabetes Care 2004 27 1047 1053 10.2337/diacare.27.5.1047 15111519

[B2] Kanaya AM Wassel CL Mathur D Stewart A Herrington D Budoff MJ Prevalence and correlates of diabetes in South asian indians in the United States: findings from the metabolic syndrome and atherosclerosis in South asians living in america study and the multi-ethnic study of atherosclerosis Metab Syndr Relat Disord 2010 8 157 164 10.1089/met.2009.0062 19943798 PMC3139526

[B3] Gholap N Davies M Patel K Sattar N Khunti K Type 2 diabetes and cardiovascular disease in South Asians Prim Care Diabetes 2011 5 45 56 10.1016/j.pcd.2010.08.002 20869934

[B4] Bindraban NR van Valkengoed IG Mairuhu G Holleman F Hoekstra JB Michels BP Prevalence of diabetes mellitus and the performance of a risk score among Hindustani Surinamese, African Surinamese and ethnic Dutch: a cross-sectional population-based study BMC Public Health 2008 8 271 10.1186/1471-2458-8-271 18673544 PMC2533321

[B5] Bos V Kunst AE Keij-Deerenberg IM Garssen J Mackenbach JP Ethnic inequalities in age- and cause-specific mortality in The Netherlands Int J Epidemiol 2004 33 1112 1119 10.1093/ije/dyh189 15166193

[B6] Mangalmurti SS Paley A Gany F Fisher EA Hochman JS South Asians and risk of cardiovascular disease: current insights and trends Ethn Dis 2010 20 474 478 21305840

[B7] Webb DR Gray LJ Khunti K Srinivasan B Taub N Campbell S Screening for diabetes using an oral glucose tolerance test within a western multi-ethnic population identifies modifiable cardiovascular risk: the ADDITION-Leicester study Diabetologia 2011 54 2237 2246 10.1007/s00125-011-2189-2 21638133

[B8] Knowler WC Barrett-Connor E Fowler SE Hamman RF Lachin JM Walker EA Reduction in the incidence of type 2 diabetes with lifestyle intervention or metformin N Engl J Med 2002 346 393 403 11832527 10.1056/NEJMoa012512 PMC1370926

[B9] Tuomilehto J Lindstrom J Eriksson JG Valle TT Hamalainen H Ilanne-Parikka P Prevention of type 2 diabetes mellitus by changes in lifestyle among subjects with impaired glucose tolerance N Engl J Med 2001 344 1343 1350 10.1056/NEJM200105033441801 11333990

[B10] Roumen C Corpeleijn E Feskens EJ Mensink M Saris WH Blaak EE Impact of 3-year lifestyle intervention on postprandial glucose metabolism: the SLIM study Diabet Med 2008 25 597 605 10.1111/j.1464-5491.2008.02417.x 18445174

[B11] Wandell PE Carlsson A Steiner KH Prevalence of diabetes among immigrants in the Nordic countries Curr Diabetes Rev 2010 6 126 133 10.2174/157339910790909404 20201798

[B12] Mason S Hussain-Gambles M Leese B Atkin K Brown J Representation of South Asian people in randomised clinical trials: analysis of trials' data BMJ 2003 326 1244 1245 10.1136/bmj.326.7401.1244 12791739 PMC161554

[B13] Hawthorne K Robles Y Cannings-John R Edwards AG Culturally appropriate health education for type 2 diabetes mellitus in ethnic minority groups Cochrane Database Syst Rev 2008:CD006424 18646153 10.1002/14651858.CD006424.pub2

[B14] Resnicow K Soler R Braithwaite RL Cultural sensitivity in substance use prevention Journal of community psychology 2000 28 271 290 10.1002/(SICI)1520-6629(200005)28:3<271::AID-JCOP4>3.0.CO;2-I

[B15] Choenni C Harmsen C Place of birth and ethnic composition of the Surinamese in the Netherlands [in Dutch] Bevolkingtrends 2007 1 74 78

[B16] Beune EJ Haafkens JA Agyemang C Schuster JS Willems DL How Ghanaian, African-Surinamese and Dutch patients perceive and manage antihypertensive drug treatment: a qualitative study J Hypertens 2008 26 648 656 10.1097/HJH.0b013e3282f4d20b 18327072

[B17] Beune EJ Haafkens JA Agyemang C Bindels PJ Inhibitors and enablers of physical activity in multiethnic hypertensive patients: qualitative study J Hum Hypertens 2010 24 280 290 10.1038/jhh.2009.61 19641519

[B18] Diagnosis and classification of diabetes mellitus Diabetes Care 2007 30 Suppl 1 S42 S47 17192378 10.2337/dc07-S042

[B19] International Expert Committee report on the role of the A1C assay in the diagnosis of diabetes Diabetes Care 2009 32 1327 1334 19502545 10.2337/dc09-9033 PMC2699715

[B20] Song Y Manson JE Tinker L Howard BV Kuller LH Nathan L Insulin sensitivity and insulin secretion determined by homeostasis model assessment and risk of diabetes in a multiethnic cohort of women: the Women's Health Initiative Observational Study Diabetes Care 2007 30 1747 1752 10.2337/dc07-0358 17468352 PMC1952235

[B21] Matthews DR Hosker JP Rudenski AS Naylor BA Treacher DF Turner RC Homeostasis model assessment: insulin resistance and beta-cell function from fasting plasma glucose and insulin concentrations in man Diabetologia 1985 28 412 419 10.1007/BF00280883 3899825

[B22] Wendel-Vos GC Schuit AJ Saris WH Kromhout D Reproducibility and relative validity of the short questionnaire to assess health-enhancing physical activity J Clin Epidemiol 2003 56 1163 1169 10.1016/S0895-4356(03)00220-8 14680666

[B23] de Munter JS van Valkengoed IG Agyemang C Kunst AE Stronks K Large ethnic variations in recommended physical activity according to activity domains in amsterdam, the netherlands Int J Behav Nutr Phys Act 2010 7 85 10.1186/1479-5868-7-85 21114828 PMC3004814

[B24] Stivoro Meetinstrumenten voor onderzoek naar roken en stoppen met roken 2006 Ref Type: Online Source

[B25] Centraal Bureau voor Statistiek Gezondheidsenquete POLS 2008 2008 Ref Type: Online Source

[B26] Dijkshoorn H van Dijk TK Janssen AP Eindrapport Amsterdamse Gezondheidsmonitor 2008 2009 Online Source, Ref Type

[B27] ROSE GA The diagnosis of ischaemic heart pain and intermittent claudication in field surveys Bull World Health Organ 1962 27 645 658 13974778 PMC2555832

[B28] Sykes K Roberts A The Chester step test-a simple yet effective tool for the prediction of aerobic capacity Physiotherapy 2004 90 183 188 10.1016/j.physio.2004.03.008

[B29] Buckley JP Sim J Eston RG Hession R Fox R Reliability and validity of measures taken during the Chester step test to predict aerobic power and to prescribe aerobic exercise Br J Sports Med 2004 38 197 205 10.1136/bjsm.2003.005389 15039259 PMC1724781

[B30] Mols F Pelle AJ Kupper N Normative data of the SF-12 health survey with validation using postmyocardial infarction patients in the Dutch population Qual Life Res 2009 18 403 414 10.1007/s11136-009-9455-5 19242822

[B31] Aaronson NK Muller M Cohen PD Essink-Bot ML Fekkes M Sanderman R Translation, validation, and norming of the Dutch language version of the SF-36 Health Survey in community and chronic disease populations J Clin Epidemiol 1998 51 1055 1068 10.1016/S0895-4356(98)00097-3 9817123

[B32] Terluin B van Marwijk HW Ader HJ de Vet HC Penninx BW Hermens ML The Four-Dimensional Symptom Questionnaire (4DSQ): a validation study of a multidimensional self-report questionnaire to assess distress, depression, anxiety and somatization BMC Psychiatry 2006 6 34 10.1186/1471-244X-6-34 16925825 PMC1590008

[B33] Claassen L Henneman L van der Weijden T Marteau T Timmermans D Being at risk for cardiovascular disease: perceptions and preventive behaviour in people with and without known genetic predisposition 2011 Amsterdam: Vrije Universiteit 10.1080/13548506.2011.644246 22360457

[B34] Health Council of the Netherlands Guidelines for a healthy diet: the ecological perspective 2011 The Hague: Health Council of the Netherlands

[B35] Kemper HCG Ooijendijk WTM Stiggelbout M Concensus over de Nederlandse norm voor gezond bewegen Tijdschrift voor Gezondheidswetenschappen (TSG) 2000 78 3 180 183

[B36] Mensink M Feskens EJM Saris WHM de Bruin TW Blaak EE Study on Lifestyle Intervention and Impaired Glucose Tolerance Maastricht (SLIM): preliminary results after one year Int J Obes Relat Metab Disord 2003 27 377 84 10.1038/sj.ijo.0802249 12629566

[B37] Kohinor MJ Stronks K Nicolaou M Haafkens JA Considerations affecting dietary behaviour of immigrants with type 2 diabetes: a qualitative study among Surinamese in the Netherlands Ethn Health 2011 16 245 258 10.1080/13557858.2011.563557 21516555

[B38] Nicolaou M van Dam RM Stronks K Acculturation and education level in relation to quality of the diet: a study of Surinamese South Asian and Afro-Caribbean residents of the Netherlands J Hum Nutr Diet 2006 19 383 393 10.1111/j.1365-277X.2006.00720.x 16961685

[B39] Greaves CJ Sheppard KE Abraham C Hardeman W Roden M Evans PH Systematic review of reviews of intervention components associated with increased effectiveness in dietary and physical activity interventions BMC Public Health 2011 11 119 10.1186/1471-2458-11-119 21333011 PMC3048531

[B40] Miller WR Rollnick S Motivational Interviewing, preparing people to change addictive behavior 1991 New York: The Guildford Press

[B41] Rubak S Sandbaek A Lauritzen T Christensen B Motivational interviewing: a systematic review and meta-analysis Br J Gen Pract 2005 55 305 312 15826439 PMC1463134

[B42] Brug J Spikmans F Aartsen C Breedveld B Bes R Fereira I Training dietitians in basic motivational interviewing skills results in changes in their counseling style and in lower saturated fat intakes in their patients J Nutr Educ Behav 2007 39 8 12 10.1016/j.jneb.2006.08.010 17276321

[B43] Broers S Smets E Bindels P Evertsz' FB Calff M De HH Training general practitioners in behavior change counseling to improve asthma medication adherence Patient Educ Couns 2005 58 279 287 10.1016/j.pec.2005.06.001 16024211

[B44] van Eijk-Hustings YJ Daemen L Schaper NC Vrijhoef HJ Implementation of Motivational Interviewing in a diabetes care management initiative in The Netherlands Patient Educ Couns 2011 84 10 15 10.1016/j.pec.2010.06.016 20638812

[B45] Schmidt M Absalah S Nierkens V Stronks K Which factors engage women in deprived neighbourhoods to participate in exercise referral schemes? BMC Public Health 2008 8 371 10.1186/1471-2458-8-371 18950533 PMC2583997

[B46] Stichting Precura Exercise on Prescription [Bewegen op Recept] 2010 Ref Type: Online Source

[B47] Hosper K Deutekom M Stronks PK The effectiveness of "Exercise on Prescription" in stimulating physical activity among women in ethnic minority groups in the Netherlands: protocol for a randomized controlled trial BMC Public Health 2008 8 406 10.1186/1471-2458-8-406 19077190 PMC2631485

[B48] Appropriate body-mass index for Asian populations and its implications for policy and intervention strategies Lancet 2004 363 157 163 14726171 10.1016/S0140-6736(03)15268-3

[B49] The Working Group on Revised Hypertension Guidelines. Techniques of office blood pressure measurement. Revised Hypertension Guidelines. 37–38 The Dutch Institute for Healthcare Improvement and the Dutch Heart Foundation 2000 Report: Ref Type

[B50] Lindstrom J Tuomilehto J The diabetes risk score: a practical tool to predict type 2 diabetes risk Diabetes Care 2003 26 725 731 10.2337/diacare.26.3.725 12610029

[B51] Prochaska JO Johnson S Lee P Shumaker SAE Schron EBE Ockene JKE The transtheoretical model of behavior change. The handbook of health behavior change 1998 2 Springer Publishing Co, New York 59 84

[B52] DiClemente CC Prochaska JO Fairhurst SK Velicer WF Velasquez MM Rossi JS The process of smoking cessation: an analysis of precontemplation, contemplation, and preparation stages of change J Consult Clin Psychol 1991 59 295 304 2030191 10.1037//0022-006x.59.2.295

[B53] Lindstrom J Eriksson JG Valle TT Aunola S Cepaitis Z Hakumaki M Prevention of diabetes mellitus in subjects with impaired glucose tolerance in the Finnish Diabetes Prevention Study: results from a randomized clinical trial J Am Soc Nephrol 2003 14 S108 S113 10.1097/01.ASN.0000070157.96264.13 12819313

[B54] Saunders RP Evans MH Joshi P Developing a process-evaluation plan for assessing health promotion program implementation: a how-to guide Health Promot Pract 2005 6 134 147 10.1177/1524839904273387 15855283

[B55] Resnicow K 1-Pass coding system for motivational interviewing: introduction and scoring. 1–7 2002 Atlana: Rollins School of Public Health, Emory University Ref Type: Report

[B56] Seeleman C Suurmond J Stronks K Cultural competence: a conceptual framework for teaching and learning Med Educ 2009 43 229 237 10.1111/j.1365-2923.2008.03269.x 19250349

[B57] Goody CM Drago L Using Cultural Competence Constructs to Understand Food Practices and Provide Diabetes Care and Education Diabetes Spectrum 2009 22

[B58] Harris-Davis E Haughton B Model for multicultural nutrition counseling competencies J Am Diet Assoc 2000 100 1178 1185 10.1016/S0002-8223(00)00342-4 11043703

[B59] Bennett CM Guo M Dharmage SC HbA(1c) as a screening tool for detection of Type 2 diabetes: a systematic review Diabet Med 2007 24 333 343 10.1111/j.1464-5491.2007.02106.x 17367307

[B60] Selvin E Steffes MW Zhu H Matsushita K Wagenknecht L Pankow J Glycated hemoglobin, diabetes, and cardiovascular risk in nondiabetic adults N Engl J Med 2010 362 800 811 10.1056/NEJMoa0908359 20200384 PMC2872990

[B61] Diagnosis and classification of diabetes mellitus Diabetes Care 2010 33 Suppl 1 S62 S69 20042775 10.2337/dc10-S062 PMC2797383

[B62] Roland M Togerson DJ Understanding controlled trials: What are pragmatic trials? BMJ 1998 316 285 10.1136/bmj.316.7127.285 9472515 PMC2665488

[B63] Douglas A Bhopal RS Bhopal R Forbes JF Gill JM Lawton J Recruiting South Asians to a lifestyle intervention trial: experiences and lessons from PODOSA (Prevention of Diabetes & Obesity in South Asians) Trials 2011 12 220 10.1186/1745-6215-12-220 21978409 PMC3201899

[B64] Ramachandran A Snehalatha C Mary S Mukesh B Bhaskar AD Vijay V The Indian Diabetes Prevention Programme shows that lifestyle modification and metformin prevent type 2 diabetes in Asian Indian subjects with impaired glucose tolerance (IDPP-1) Diabetologia 2006 49 289 297 16391903 10.1007/s00125-005-0097-z

[B65] Bartlett C Doyal L Ebrahim S Davey P Bachmann M Egger M The causes and effects of socio-demographic exclusions from clinical trials Health Technol Assess 2005 9 iii x 16181564 10.3310/hta9380

[B66] El FF Bruijnzeels MA Foets MM Hoes AW Different distribution of cardiovascular risk factors according to ethnicity: a study in a high risk population J Immigr Minor Health 2008 10 559 565 10.1007/s10903-008-9144-4 18483765

[B67] Greenland P Hildreth NG Maiman LA Attendance patterns and characteristics of participants in public cholesterol screening Am J Prev Med 1992 8 159 164 1633003

[B68] Blackwell CS Foster KA Isom S Katula JA Vitolins MZ Rosenberger EL Healthy Living Partnerships to Prevent Diabetes: recruitment and baseline characteristics Contemp Clin Trials 2011 32 40 49 10.1016/j.cct.2010.10.006 20974289 PMC3005835

[B69] Claassen L Henneman L Nijpels G Dekker J Marteau T Timmermans D Perceived control over diabetes risk and preventive behaviour: the role of family history and self-malleability 2011 Amsterdam: Vrije Universiteit 73 82

[B70] Shephard RJ Limits to the measurement of habitual physical activity by questionnaires Br J Sports Med 2003 37 197 206 10.1136/bjsm.37.3.197 12782543 PMC1724653

[B71] Kremers SP Visscher TL Seidell JC Van MW Brug J Cognitive determinants of energy balance-related behaviours: measurement issues Sports Med 2005 35 923 933 10.2165/00007256-200535110-00001 16271007

