# Supplementary material for: Feasibility and effectiveness of a targeted diabetes prevention program for 18 to 60-year-old South Asian migrants: design and methods of the DH!AAN study
Source: BMC Public Health. 2012 May 23;12:371. doi: 10.1186/1471-2458-12-371 (PMC3504520; doi:10.1186/1471-2458-12-371)
Supplement: Additional file 1 — Online supplement. Changes to the original protocol of the randomized controlled trial [2,8,36,53,64]. [file 1471-2458-12-371-S1.doc]

**Online Supplement.** Changes to the original protocol of the randomized controlled trial

| **Subject** | **Original protocol** | **Current protocol** |
| --- | --- | --- |
| Study duration | Total duration is 4 years:  - 1 year of preparation and recruitment  - 3 years of follow-up, with measurements at baseline and after 1 and 3 years | Total duration is 4 years:  - 2 years of preparation and recruitment  - 2 years of follow-up, with measurements at baseline and after 1 and 2 years |
| Primary outcome | - Incidence of type 2 diabetes mellitus | Weight, mean glucose level, and behavior (physical activity and diet) |
| Inclusion criteria | - IFG and/or IGT^a^ during both initial screening and baseline measurement  - No family members or volunteers | - IFG and/or IGT^a,b^, or a Hb_A1c_ of ≥ 6.0% and/or a HOMA-IR ≥ 2.39 mmol/mol during initial screening and no diabetes^c^ during baseline measurement  - Family members and volunteers allowed |
| Home visits | - | Home visits to be offered to those unwilling or unable to visit the study clinic for measurements after 1 and 2 years |

Legend:

^a^FPG of 100–125 mg/dL (5.6 – 6.9 mmol/L) and/or 2-h postload glucose of 140–199 mg/dL (7.8–11.0 mmol/L)

^b^IGT only among participants until April 19, 2010

^c^FPG ≥ 126 mg/dL (7.0 mmol/L) and/or 2-h plasma glucose ≥ 200 mg/dL (11.1 mmol/L)

IFG, impaired fasting glucose; IGT, impaired glucose tolerance; FPG, fasting plasma glucose; Hb_A1c_, glycated hemoglobin; HOMA-IR, homeostasis model assessment of estimated insulin resistance

# Original power calculation

We calculated the power to determine what size of study population we needed to identify a significant difference in incidence of type 2 diabetes (DM) after 3 years. To achieve a risk reduction of 50% (other studies reached a risk reduction of 28–58%), we needed 6000 invitees, including 500 people with pre-diabetes (250 people per trial group).We assumed a power of 80%, an alpha of 5%, and a dropout rate of 20% for the calculation [8,24,52,63]. We assume that the 3-year cumulative incidence of DM among people with IFG and/or IGT is 25% (20–55%, [8,24,52,63]) and that the prevalence of IFG and/or IGT among South Asian migrants is 35–40% [2; pilot study, unpublished data]. We expect 85% of the screening participants to be potentially eligible, with a response rate of 25% and a participation rate of 60% for the trial [pilot study, unpublished data].
